# Modified Gegen Qinlian Decoction Ameliorates DSS-Induced Colitis in Mice via the Modulation of NF-*κ*B and Nrf2/HO-1 Pathways

**DOI:** 10.1155/mi/7468297

**Published:** 2025-01-16

**Authors:** Jinke Huang, Jiaqi Zhang, Zhihong Liu, Jing Ma, Yifan Wang, Fengyun Wang, Xudong Tang

**Affiliations:** ^1^Department of Gastroenterology, The Second Affiliated Hospital of Guangzhou University of Chinese Medicine (Guangdong Provincial Hospital of Chinese Medicine), Guangzhou, China; ^2^Institute of Digestive Diseases, Xiyuan Hospital of China Academy of Chinese Medical Sciences, Beijing, China

**Keywords:** inflammation, modified Gegen Qinlian decoction, oxidative stress, ulcerative colitis

## Abstract

**Background:** This study aims to reveal the potential molecular mechanisms of modified Gegen Qinlian decoction (MGQD) in relieving ulcerative colitis (UC).

**Methods:** C57BL/6J mice were used to establish experimental colitis via dextran sodium sulfate (DSS). Body weight, disease activity index (DAI), spleen weight, colon length, and histopathologic features were measured to evaluate the therapeutic effects of MGQD on mice with UC. The ELISA kits were employed to assess the concentrations of interleukin (IL)-6, IL-1β, tumor necrosis factor-α (TNF-α), glutathione (GSH), reactive oxygen species (ROS), and malondialdehyde (MDA). Western blot analyses were used to assess the levels of IκBα, p65, p-IκBα, p-p65, HO-1, and Nrf2. Moreover, the protein levels of Nrf2 and p-p65 were assessed by immunofluorescence.

**Results:** Colitis-related symptoms in mice were significantly alleviated by MGQD. Moreover, MGQD inhibited the levels of TNF-α, IL-1β, IL-6, MDA, and ROS and increased the level of GSH in mice with UC. Mechanistically, MGQD prevented the activation of the NF-κB pathway and concomitantly promoted the activation of the Nrf2/HO-1 pathway.

**Conclusion:** MGQD alleviated UC by suppressing inflammation and oxidative stress via the modulation of NF-κB and Nrf2/HO-1 pathways, suggesting that MGQD may be a candidate therapy for UC.

## 1. Introduction

Ulcerative colitis (UC) is characterized by the recurrent and widespread mucosal inflammation [1]. The prevalence and incidence of UC have been increasing globally [2]. Typical symptoms of UC are bloody diarrhea, abdominal pain, and rectal bleeding, which greatly reduce the patients' quality of life and increase their financial burden [3]. The disruption of epithelial barrier and abnormal inflammatory responses has long been considered to be responsible for the development and progression of UC [4]. Treatments for UC include 5-aminosalicylic acid (5-ASA), corticosteroids, biologics (e.g., anti-integrins and anticytokines), thiopurines, and small molecules (sphingosine-1-phosphate receptor modulators and Janus kinase inhibitors) [5]. Although the therapeutic options are expanding, patients with UC are not always satisfied with the outcome of their treatment, and 10%–20% of patients still require proctocolectomy for medically refractory disease [1]. Therefore, research into complementary and alternative therapies for the treatment of UC is of great interest [6].

Gegen Qinlian decoction, which is composed of *Glycyrrhiza uralensis* Fisch, *Coptis chinensis* French, *Scutellaria baicalensis* Georgi, and *Pueraria lobata* (Willd.) Ohwi [7], is beneficial in the treatment of UC [8]. Under the guidance of clinical practice and Chinese medicine theory, we have added *Zingiber officinale* Roscoe and *Talcum* to Gegen Qinlian decoction, thus formulating modified Gegen Qinlian decoction (MGQD) [9–11]. Our previous studies [9–11] revealed that MGQD can effectively alleviate UC, but the specific mechanisms remain poorly understood. Therefore, this study aimed to reveal the molecular mechanisms by which MGQD ameliorates UC through a mouse model of colitis.

## 2. Materials and Methods

### 2.1. Preparation of MGQD

MGQD (*G. uralensis* Fisch [6 g], *C. chinensis* French [9 g], *Scutellaria baicalensis* Georgi [9 g], *P. lobata* (Willd.) Ohwi [24 g], *Z. officinale* Roscoe [9 g], and *Talcum* [9 g]) was provided by the pharmacy of Xi Yuan Hospital. *Talcum* was decocted for 30 min, and then the other herbs were added and decocted for 1.5 h and finally filtered. The filtered residue was added to six times the volume of distilled water and boiled again for 1 h and then filtered. The filtrate obtained on both occasions was mixed and concentrated to 0.5, 1, and 2 g/mL of drug, respectively.

### 2.2. Experimental Protocol for Mice

**Figure 1:**
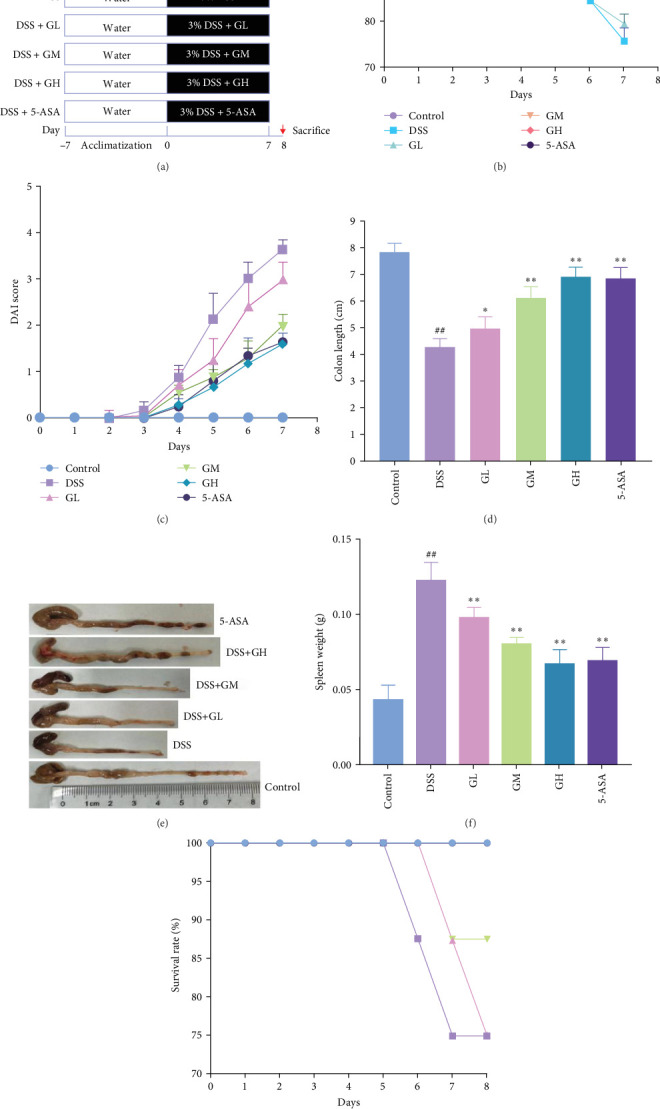
MGQD alleviates the symptoms in DSS-induced colitis. (A) Experimental protocol for mice. (B) Daily changes in body weight during DSS-induced period (*n* = 6). (C) DAI during DSS-induced period (*n* = 6). (D, E) Changes of colon length in different groups (*n* = 6). (F) Changes of spleen weight in different groups (*n* = 6). (G) survival rate of mice in each group (*n* = 8). ##*p* < 0.005 versus control group; *⁣*^*∗*^*p* < 0.05, *⁣*^*∗∗*^*p* < 0.005 versus DSS group. 5-ASA, 5-aminosalicylic acid; DAI, disease activity index; DSS, dextran sulfate sodium; GH, high-dose MGQD; GL, low-dose MGQD; GM, medium-dose MGQD; MGQD, modified Gegen Qinlian decoction.

Male C57BL/6J mice (6–8 weeks old) were purchased from SPF Biotechnology Co., Ltd. (license number: 2019-0010, Beijing, China). Mice were housed in the experimental animal center of Xiyuan Hospital (12/12 h light/dark cycle, room temperature 24 ± 1°C) with free access to food and water. Mice were randomly assigned to the groups of control, dextran sulfate sodium (DSS), 5-ASA, high-dose MGQD (GH), medium-dose MGQD (GM), and low-dose MGQD (GL), with *n* = 8 mice per group. Acute colitis was induced by adding 3% DSS to the drinking water of all mice except the control group. Meanwhile, mice in the GL, GM, and GH groups were given 5, 10, and 20 g/kg MGQD by gavage once daily for 7 days according to the previous protocol [9–11]. For mice in the 5-ASA group, 0.1 g/kg dose of 5-ASA was intragastrically administered once a day for 7 days. In addition, the equal volume of saline gavage was administered to the DSS and control groups. The experimental procedure is presented in Figure 1A.

### 2.3. Disease Activity Index (DAI)

Body weight, fecal consistency, and blood stools were measured daily in mice, and DAI scores were calculated according to previously reported methods (Table 1) [12].

### 2.4. Histological Analysis

Following 4% paraformaldehyde fixation, the acquired colon tissue samples were paraffin embedded. Before hematoxylin and eosin (H&E) staining, the paraffin-embedded tissue blocks were sliced to 5-µm-thick sections. Sections were scored for histopathology according to previously published criteria (Table 2) [12].

### 2.5. ELISA Analysis

The ELISA kits were employed to assess the concentrations of interleukin (IL)-6 (Ruixin Biotechnology, RX203049M), IL-1β (Ruixin Biotechnology, RX302869R), tumor necrosis factor-α (TNF-α) (Ruixin Biotechnology, RX202412M), glutathione (GSH) (Ruixin Biotechnology, RX202918M), reactive oxygen species (ROS) (Ruixin Biotechnology, RXSH0666), and malondialdehyde (MDA) (Ruixin Biotechnology, RXWB0005) in the colonic tissue samples of mice in accordance with the instructions provided by the manufacturer.

### 2.6. Immunofluorescence

The colon tissue was fixed with 4% paraformaldehyde, paraffin-embedded, and sectioned at 5 μm. Tissue slides were cleaned with deionized water after being dewaxed with xylene and rehydrated with gradient ethanol. Antigenic repair techniques were carried out employing microwaves and antigenic repair solutions. The slides were sealed with 10% normal goat serum for 1 h at room temperature. They were then incubated with primary antibodies (Nrf2 [Proteintech, 80593-1-RR] and p-p65 [Proteintech, 82335-1-RR]) at 4°C overnight before being stained with fluorescent (CY3) or fluorescent (FITC)-labeled secondary antibodies. Following the second antibody incubation, the tablets were stained with DAPI solution and sealed with a sealing solution containing an antifluorescence quenching agent. All operations were performed in dark conditions. Caseviewer and Leica LAS image acquisition systems were applied to observe sections.

### 2.7. Western Blot Analysis

Proteins were isolated from colon tissue samples of mice and quantified. After being separated into proteins of various molecular weights using gel electrophoresis, the proteins were transferred to polyvinylidene difluoride membranes and blocked with skim milk. Then, membranes were mixed with IκBα (Proteintech, 10268-1-AP), p65 (Proteintech, 10745-1-AP), p-IκBα (Proteintech, 82349-1-RR), p-p65 (Proteintech, 82335-1-RR), HO-1 (Proteintech, 27282-1-AP), and Nrf2 (Proteintech, 80593-1-RR) primary antibodies, respectively, and incubated at 4°C overnight. The samples were then treated for 2 h at room temperature with HRP-labeled rabbit or mouse IgG secondary antibodies. ECL solution (P0018, Beyotime) was used to visualize the protein signals.

### 2.8. Statistical Analysis

The mean ± standard error of the mean was used to express all data. For comparisons between two or more groups, one-way analysis of variance was employed. All statistical analyses were carried out by SPSS 26.0 and GraphPpad Prism version 9 software. A statistically significant difference was defined as *p* < 0.05.

## 3. Results

### 3.1. MGQD Alleviated the Symptoms of Mice with UC

In comparison to the control group, mice elicited by DSS displayed evident indications of colitis, such as weight alleviation, bloody stools, loose stools, shortened colon length, and increased spleen weight (*p* < 0.05, Figure 1). Interestingly, mice treated with MGQD and 5-ASA recovered their body weight and colon length and had significantly lower DAI scores and spleen weight compared to the DSS group (*p* < 0.05, Figure 1B–F). In addition, the mortality rate of DSS-induced colitis mice was as high as 25% compared with that of mice in the control group (*p* < 0.05, Figure 1G). Compared with mice in the DSS group, the mortality rate of mice treated with MGQD was significantly reduced (*p* < 0.05), with the survival rate of mice in the GH group reaching 100% (Figure 1G). Notably, MGQD showed a dose-dependent improvement of symptoms associated with colitis, and the anticolitis effect of MGQD at 20 g/kg was similar to that of 5-ASA (*p* > 0.05).

### 3.2. MGQD Alleviated the Pathology of Mice with UC

**Figure 2:**
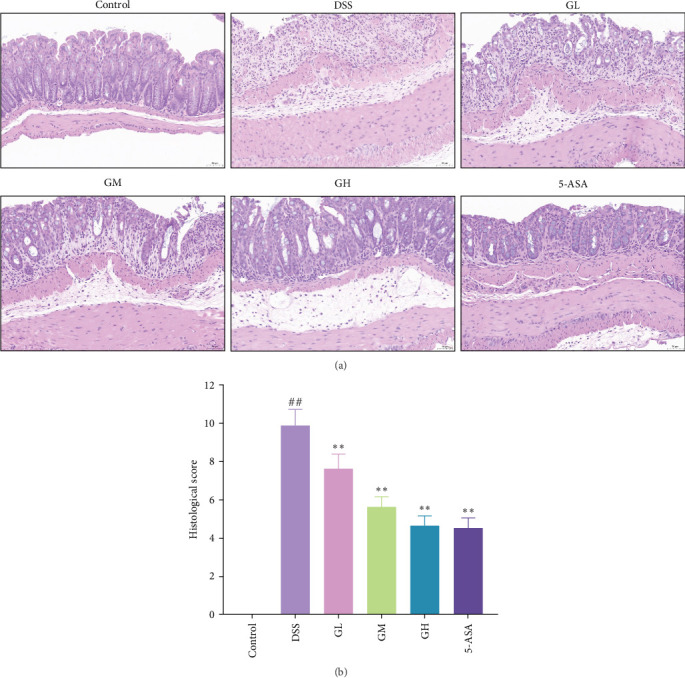
MGQD prevented colon damage in mice with UC. (A) Representative images of hematoxylin and eosin (H&E)-stained colon tissue. Scale bar = 50 µm (*n* = 6). (B) Comparison of histopathological score in different groups (*n* = 6). ##*p* < 0.005 versus control group; *⁣*^*∗∗*^*p* < 0.005 versus DSS group. 5-ASA, 5-aminosalicylic acid; DSS, dextran sulfate sodium; GH, high-dose MGQD; GL, low-dose MGQD; GM, medium-dose MGQD; MGQD, modified Gegen Qinlian decoction; UC, ulcerative colitis.

In comparison to the control group, mice with UC exhibited mucosal injury, infiltration of inflammatory cells, goblet cell loss, abnormal crypts, and higher histopathologic scores (*p* < 0.05, Figure 2). Interestingly, pathological changes and histopathological scores of colonic tissues were significantly improved in mice treated with MGQD and 5-ASA compared to the DSS group (*p* < 0.05, Figure 2).

### 3.3. MGQD Inhibited Cytokine Production and Oxidative Stress

**Figure 3:**
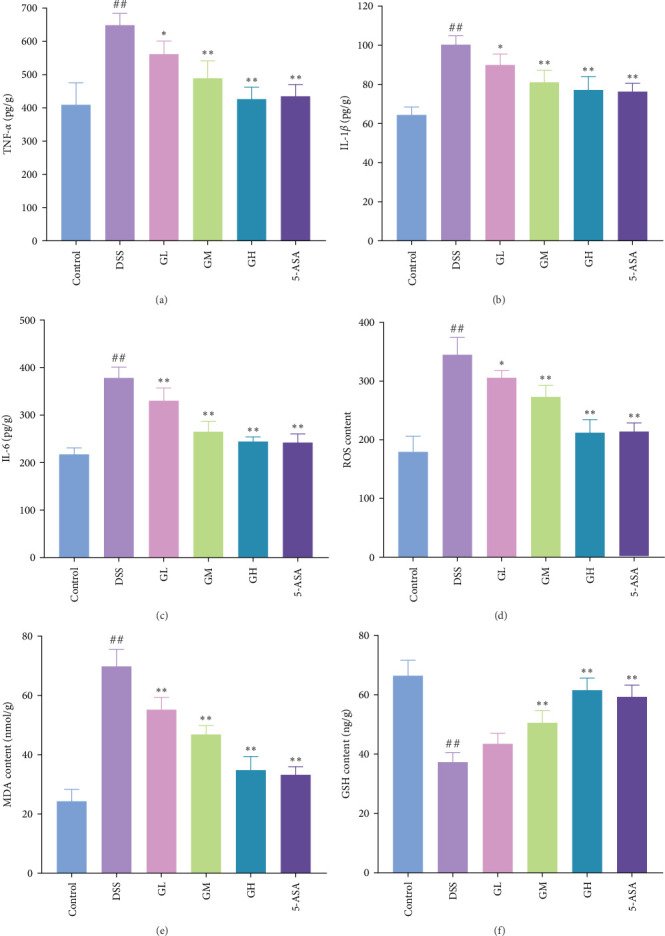
MGQD inhibited inflammatory cytokine levels and oxidative stress in mice with UC. (A) The level of TNF-α in different groups (*n* = 6). (B) The level of IL-1β in different groups (*n* = 6). (C) The level of IL-6 in different groups (*n* = 6). (D) The level of ROS in different groups (*n* = 6). (E) The level of MDA in different groups (*n* = 6). (F) The level of GSH in different groups (*n* = 6). ## *p* < 0.005 versus control group; *⁣*^*∗*^*p* < 0.05, *⁣*^*∗∗*^*p* < 0.005 versus DSS group. GSH, glutathione; IL, interleukin; MDA, malondialdehyde; MGQD, modified Gegen Qinlian decoction; ROS, reactive oxygen species; TNF-α, tumor necrosis factor-α; UC, ulcerative colitis.

In comparison to the control group, the colonic tissues of mice with UC showed considerably higher expression levels of the proinflammatory cytokines, including IL-1β, IL-6 and TNF-α (*p* < 0.05, Figure 3). Interestingly, MGQD and 5-ASA treatment significantly reversed elevated proinflammatory cytokine levels in mice with UC (*p* < 0.05, Figure 3).

**Figure 4:**
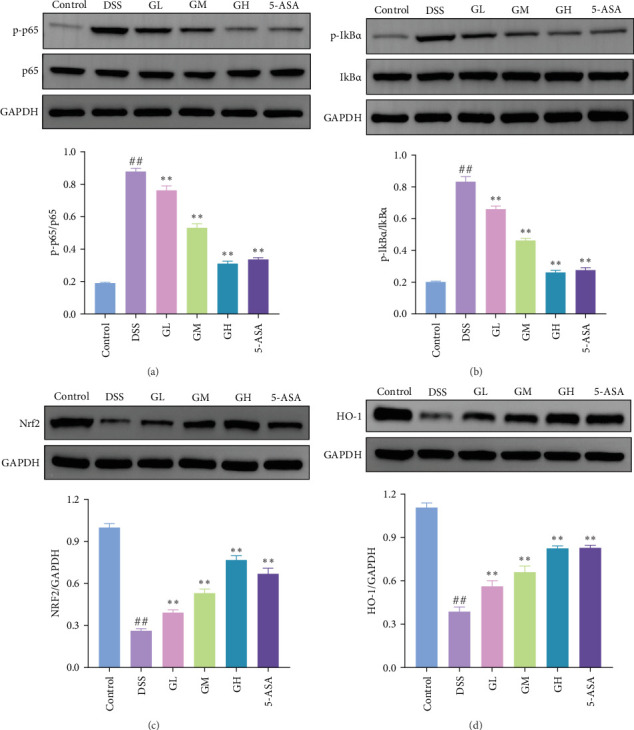
MGQD alleviated DSS-induced colitis by modulating Nrf2/HO-1 and NF-κB pathways. (A) Representative immunoblots and relative expression level of Nrf2 protein (*n* = 5). (B) Representative immunoblots and relative expression level of HO-1 protein (*n* = 5). (C) Representative immunoblots and relative expression levels of p65 and p-p65 proteins (*n* = 5). (D) Representative immunoblots and relative expression levels of IκBα and p-IκBα proteins (*n* = 5). ## *p* < 0.005 versus control group; *⁣*^*∗∗*^*p* < 0.005 versus DSS group. 5-ASA, 5-aminosalicylic acid; DSS, dextran sulfate sodium; GH, high-dose MGQD; GL, low-dose MGQD; GM, medium-dose MGQD; MGQD, modified Gegen Qinlian decoction.

In comparison to the control group, the colonic tissues of mice with UC showed considerably higher levels of ROS and MDA while lower level of GSH (*p* < 0.05, Figure 4). Interestingly, MGQD and 5-ASA therapies significantly reversed the abnormal alterations in these metrics in mice with UC (*p* < 0.05, Figure 3).

### 3.4. MGQD Inhibited the NF-κB Pathway and Activated the Nrf2/HO-1 Pathway in Mice With UC

**Figure 5:**
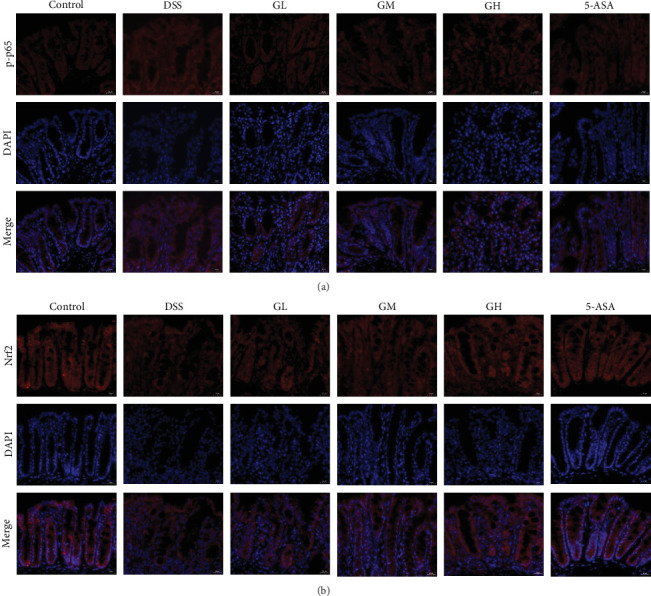
MGQD modulated the nuclear translocation of p-p65 and Nrf2 in colon tissue of mice with UC. (A) Representative immunofluorescence images showing the expression level of p-p65 protein (*n* = 5, scale bar = 20 μm). (B) Representative immunofluorescence images showing the expression level of Nrf2 protein (*n* = 5, scale bar = 20 μm). ## *p* < 0.005 versus control group; *⁣*^*∗∗*^*p* < 0.005 versus DSS group. 5-ASA, 5-aminosalicylic acid; DSS, dextran sulfate sodium; GH, high-dose MGQD; GL, low-dose MGQD; GM, medium-dose MGQD; MGQD, modified Gegen Qinlian decoction; UC, ulcerative colitis.

The NF-κB pathway was activated in the colonic tissue of mice with UC, as evidenced by considerably increased phosphorylation protein expression of p65 and IκBα when compared to that of mice in the control group (*p* < 0.05, Figure 4A–B). Interestingly, the elevated levels of p-p65 and p-IκBα in the colonic tissues of mice with UC treated with MGQD and 5-ASA were significantly reversed (*p* < 0.05, Figure 4A–B). In line with this, the results of immunofluorescence assay showed that p-p65 was significantly inhibited by MGQD and 5-ASA treatment in mice with UC (Figure 5A).

The Nrf2/HO-1 pathway was suppressed in the colonic tissue of mice with UC, as evidenced by considerably decreased protein expression of Nrf2 and HO-1 when compared to that of mice in the control group (*p* < 0.05, Figure 4C–D). Interestingly, the decreased levels of Nrf2 and HO-1 in the colonic tissues of mice with UC treated with MGQD and 5-ASA were significantly reversed (*p* < 0.05, Figure 4C–D). In line with this, immunofluorescence assay showed that MGQD and 5-ASA significantly upregulated the expression of Nrf2 in mice with UC (Figure 5B).

## 4. Discussion

UC has been characterized by inflammation of the mucosa [13]. TNF-α and ILs are important mediators of inflammatory reactions in UC [14]. Fundamental to the regulation of inflammation, NF-κB is a key transcriptional regulator of innate and adaptive immunity that regulates multiple signaling pathways and the generation of proinflammatory cytokines. UC and experimental intestinal inflammation models including DSS, trinitrobenzene sulfonic acid-induced colitis, and IL-10 knockout mice are characterized by NF-κB activation and increased expression of proinflammatory NF-κB target genes [15, 16]. When activated, NF-κB complex translocates to the nucleus, where it induces the expressions of proinflammatory genes including those of cytokines and chemokines [17]. Thus, the activation of NF-κB is known to be extremely important in the pathogenesis of UC and has been proposed as a major culprit and therapeutic target for UC [18]. Targeted inhibition of the NF-κB pathway has been shown to be a therapeutic strategy with ameliorative effects on UC. By targeting the NF-κB pathway, a variety of natural substances have been found to alleviate intestinal inflammation [19, 20]. Similarly, we also observed the suppression of NF-κB pathway and the decrease of proinflammatory cytokines levels in UC mice intervened by MGQD in the present study, suggesting a mechanism by which MGQD alleviates colonic inflammatory injury by inhibiting the NF-κB pathway.

Accumulating evidence suggests that oxidative stress plays an important role in triggering an inflammatory response and inducing UC pathogenesis, which impacts on the progression of the disease in multiple ways [21]. Nrf2 is an important transcriptional regulator involved in redox homeostasis and has an irreplaceable role in promoting antioxidant responses in organisms [22]. Among the genes upregulated by Nrf2, HO-1 has received significant attention because the products of HO-1-induced heme catabolism have well-recognized antioxidant and anti-inflammatory properties [23]. Evidence for the involvement of Nrf2/HO-1 pathway in the course of UC was first published in 2006, when it was reported that DSS-induced colitis was associated with increased expression of Nrf2 regulatory enzymes and that the increased susceptibility of Nrf2-deficient mice to DSS-induced colitis may be due to decreased expression of HO-1 as well as increased expression of proinflammatory mediators [24]. In addition, the levels of proinflammatory cytokines and lipid peroxides were significantly increased in the colon of Nrf2 knockout mice, whereas the expression levels of antioxidant enzymes were decreased [25]. Therefore, the Nrf2/HO-1 pathway may represent a promising avenue to treat UC, and the mechanism of action of targeting this pathway involves regulating oxidative stress, reducing intestinal inflammation, repairing the intestinal mucosal barrier, and preventing ferroptosis in intestinal epithelial cells [26]. By targeting the Nrf2/HO-1 pathway, several natural substances with therapeutic potential for UC have been identified [26]. Similarly, we also observed the activation of Nrf2/HO-1 pathway and the decrease of oxidative stress index in UC mice intervened by MGQD in the present study, suggesting a mechanism by which MGQD suppresses oxidative stress by activating the Nrf2/HO-1 pathway.

Limitations should be acknowledged. First, our previous study [9] identified aicalin, puerarin, palmatine chloride, wogonin, and berberine chloride as the major components of MGQD by high-performance liquid chromatography, but we have not explored the effects of the individual components of MGQD on UC in isolation, and therefore, we were unable to identify the specific components that have a mitigating effect on UC. Second, the signaling pathways associated with UC are multiple, and MGQD may have multiple targets in the treatment of UC; further studies to explore other pathways involved in the treatment of UC with MGQD are still necessary.

## 5. Conclusion

MGQD alleviated UC by suppressing inflammation and oxidative stress via the modulation of NF-κB and Nrf2/HO-1 pathways, suggesting that MGQD may be a candidate therapy for UC.

## Figures and Tables

**Table 1:** DAI scoring system.

Score	Weight loss	Stool consistency	Occult/gross rectal bleeding
0	None	Normal	Normal
1	1%–5%	—	—
2	5%–10%	Loose stools	Hemoccult+
3	10%–20%	—	—
4	>20%	Diarrhea	Gross bleeding

*Note:* The clinical criteria used to evaluate the grade of extent of intestinal inflammation. Scores were tallied for each category and then divided by three to obtain the DAI.

Abbreviation: DAI, disease activity index.

**Table 2:** Histological colitis scoring system.

Score	Inflammation severity	Inflammation extent	Crypt damage	Percent involvement
0	None	None	None	0%
1	Mild	Mucosa	Basal 1/3 damage	1%–25%
2	Moderate	Submucosa	Basal 2/3 damage	26%–50%
3	Severe	Transmural	Crypt lost; surface epithelium present	51%–75%
4	—	—	Crypt and surface epithelium lost	76%–100%

*Note:* The score ranges from 0 to 14 (total score), which represents the sum of scores from 0 to 4 for severity and extent of inflammation, crypt damage, and percent of the colon involved. All evaluations were performed by observers unaware of the treatment groups.

## Data Availability

The datasets generated for this study are available upon request to the corresponding author.

## Nomenclature

UC: Ulcerative colitis
MGQD: Modified Gegen Qinlian decoction
DSS: Dextran sulfate sodium
ROS: Reactive oxygen species
GSH: Glutathione
MDA: Malondialdehyde
5-ASA: 5-Aminosalicylic acid
GM: Medium-dose MGQD
GL: Low-dose MGQD
GH: High-dose MGQD
H&E: Hematoxylin and eosin
DAI: Disease activity index.

## References

[1] de Souza H. S. P., Fiocchi C. (2016). Immunopathogenesis of IBD: Current State of the Art. *Nature Reviews Gastroenterology & Hepatology*.

[2] Retnakumar S. V., Muller S. (2019). Pharmacological Autophagy Regulators as Therapeutic Agents for Inflammatory Bowel Diseases. *Trends in Molecular Medicine*.

[3] Neurath M. F. (2019). Targeting Immune Cell Circuits and Trafficking in Inflammatory Bowel Disease. *Nature Immunology*.

[4] Le Berre C., Honap S., Peyrin-Biroulet L. (2023). Ulcerative Colitis. *The Lancet*.

[5] de Mattos B. R. R., Garcia M. P. G., Nogueira J. B. (2015). Inflammatory Bowel Disease: An Overview of Immune Mechanisms and Biological Treatments. *Mediators of Inflammation*.

[6] Picardo S., Altuwaijri M., Devlin S. M., Seow C. H. (2020). Complementary and Alternative Medications in the Management of Inflammatory Bowel Disease. *Therapeutic Advances in Gastroenterology*.

[7] Lu J.-Z., Ye D., Ma B.-L. (2021). Constituents, Pharmacokinetics, and Pharmacology of Gegen-Qinlian Decoction. *Frontiers in Pharmacology*.

[8] Huang J., Zhang J., Wang Y. (2022). Scientific Evidence of Chinese Herbal Medicine (Gegen Qinlian Decoction) in the Treatment of Ulcerative Colitis. *Gastroenterology Research and Practice*.

[9] Wang Y., Zhang J., Xu L. (2021). Modified Gegen Qinlian Decoction Regulates Treg/Th17 Balance to Ameliorate DSS-Induced Acute Experimental Colitis in Mice by Altering the Gut Microbiota. *Frontiers in Pharmacology*.

[10] Ma J., Zhang J., Wang Y. (2023). Modified Gegen Qinlian Decoction Ameliorates DSS-Induced Chronic Colitis in Mice by Restoring the Intestinal Mucus Barrier and Inhibiting the Activation of *γ*δT17 Cells. *Phytomedicine*.

[11] Huang J., Zhang J., Liu Z., Ma J., Wang F., Tang X. (2024). Modified Gegen Qinlian Decoction Ameliorates DSS-Induced Ulcerative Colitis in Mice by Inhibiting Ferroptosis via Nrf2/GPX4 Pathway. *Journal of Food Quality*.

[12] Kihara N., de la Fuente S. G., Fujino K., Takahashi T., Pappas T. N., Mantyh C. R. (2003). Vanilloid Receptor-1 Containing Primary Sensory Neurones Mediate Dextran Sulphate Sodium Induced Colitis in Rats. *Gut*.

[13] Cleveland N. K., Torres J., Rubin D. T. (2022). What Does Disease Progression Look Like in Ulcerative Colitis, and How Might It Be Prevented?. *Gastroenterology*.

[14] Nakase H., Sato N., Mizuno N., Ikawa Y. (2022). The Influence of Cytokines on the Complex Pathology of Ulcerative Colitis. *Autoimmunity Reviews*.

[15] Elson C. O., Cong Y., McCracken V. J., Dimmitt R. A., Lorenz R. G., Weaver C. T. (2005). Experimetnal Models of Inflammatory Bowel Disease Reveal Innate, Adaptive, and Regulatory Mechanisms of Host Dialogue With the Microbiota. *Immunological Reviews*.

[16] Lorenz R. G., McCracken V. J., Elson C. O. (2005). Animal Models of Intestinal Inflammation: Ineffective Communication Between Coalition Members. *Springer Seminars in Immunopathology*.

[17] Lawrence T. (2009). The Nuclear Factor NF-*κ*B Pathway in Inflammation. *Cold Spring Harbor Perspectives in Biology*.

[18] Jobin C., Sartor B. R. (2000). NF-*κ*B Signaling Proteins as Therapeutic Targets for Inflammatory Bowel Diseases. *Inflammatory Bowel Diseases*.

[19] Hou J., Hu M., Zhang L., Gao Y., Ma L., Xu Q. (2021). Dietary Taxifolin Protects Against Dextran Sulfate Sodium-Induced Colitis via NF-*κ*B Signaling, Enhancing Intestinal Barrier and Modulating Gut Microbiota. *Frontiers in Immunology*.

[20] Zhu W., Ren L., Zhang L., Qiao Q., Farooq M. Z., Xu Q. (2020). The Potential of Food Protein-Derived Bioactive Peptides against Chronic Intestinal Inflammation. *Mediators of Inflammation*.

[21] Piotrowska M., Swierczynski M., Fichna J., Piechota-Polanczyk A. (2021). The Nrf2 in the Pathophysiology of the Intestine: Molecular Mechanisms and Therapeutic Implications for Inflammatory Bowel Diseases. *Pharmacological Research*.

[22] Ngo V., Duennwald M. L. (2022). Nrf2 and Oxidative Stress: A General Overview of Mechanisms and Implications in Human Disease. *Antioxidants*.

[23] O’Rourke S. A., Shanley L. C., Dunne A. (2024). The Nrf2-HO-1 System and Inflammaging. *Frontiers in Immunology*.

[24] Khor T. O., Huang M. T., Kwon K. H., Chan J. Y., Reddy B. S., Kong A. N. (2006). Nrf2-Deficient Mice Have an Increased Susceptibility to Dextran Sulfate Sodium–Induced Colitis. *Cancer Research*.

[25] Osburn W. O., Karim B., Dolan P. M. (2007). Increased Colonic Inflammatory Injury and Formation of Aberrant Crypt Foci in Nrf2-Deficient Mice upon Dextran Sulfate Treatment. *International Journal of Cancer*.

[26] Yuan L., Wang Y., Li N. (2024). Mechanism of Action and Therapeutic Implications of Nrf2/HO-1 in Inflammatory Bowel Disease. *Antioxidants*.

